# Transcytosis of HIV-1 through Vaginal Epithelial Cells Is Dependent on Trafficking to the Endocytic Recycling Pathway

**DOI:** 10.1371/journal.pone.0096760

**Published:** 2014-05-15

**Authors:** Ballington L. Kinlock, Yudi Wang, Tiffany M. Turner, Chenliang Wang, Bindong Liu

**Affiliations:** 1 Center for AIDS Health Disparities Research, Meharry Medical College, Nashville, Tennessee, United States of America; 2 Department of Microbiology and Immunology, Meharry Medical College, Nashville, Tennessee, United States of America; 3 Institute of Gastroenterology and Institute of Human Virology, Sun Yat-sen University, Guangzhou, Guangdong, Peoples of Republic of China; German Primate Center, Germany

## Abstract

**Background:**

While it is accepted that viruses can enter epithelial cells by endocytosis, the lack of an established biological mechanism for the trafficking of infectious virions through vaginal epithelial cells and their release from the plasma membrane has contributed to ongoing controversy about whether endocytosis is a mere artifact of some cell culture systems and whether squamous vaginal epithelial cells are even relevant as it pertains to HIV-1 transmission.

**Methodology/Principal Findings:**

In this study, we investigated the intracellular trafficking pathway that HIV-1 exploits to transcytose vaginal epithelial cells. The reduction of endosome tubulation by recycling endosome inhibitors blocked transcytosis of HIV-1 in a cell culture and transwell system. In addition, we demonstrate that although heat-inactivated virus was endocytosed as efficiently as native virus, heat-inactivated virus was trafficked exclusively to the lysosomal pathway for degradation following endocytosis. Lysosomal protease-specific inhibitors blocked the degradation of inactivated virions. Immunofluorescence analysis not only demonstrated that HIV-1 was inside the cells but the different colocalization pattern of native vs. heat inactivated virus with transferrin provided conclusive evidence that HIV-1 uses the recycling pathway to get across vaginal epithelial cells.

**Conclusions/Significance:**

Altogether, our findings demonstrate the precise intracellular trafficking pathway utilized by HIV-1 in epithelial cells, confirms that HIV-1 transcytosis through vaginal epithelial cells is a biological phenomenon and brings to light the differential intracellular trafficking of native vs heat-inactivated HIV-1 which with further exploration could prove to provide valuable insights that could be used in the prevention of transcytosis/transmission of HIV-1 across the mucosal epithelia.

## Introduction

Transcytosis is a transport mechanism commonly utilized by numerous cell types for the transport of various cargo [1]. The cargo moved by this process is immensely diverse, and the specific pathways involved depend on both the particular cellular context and the cargo being transported [1]. The process by which cargo is directed to a transcytotic pathway instead of the lysosomal degradation pathway is not completely understood, but seems to be dependent on the nature of the cargo in question [1], [2].

Conflicting reports has led to questions about whether transcytosis of HIV-1 is a mere artifact of some cell culture systems. While some reports suggests that transcytosis of HIV-1 through female genital epithelial cells does not occur [3]–[5], data from other studies have demonstrated that transcytosis of HIV-1 across cervical, intestinal, adult penile urethra and other epithelial layer does occur [6]–[11], with cell-associated HIV-1 being transcytosed more efficiently compared to cell-free virus [7]. The mechanism as to why cell-associated virus transcytose at a higher rate compared to cell free virus is not completely understood but it has been suggested that HIV-1 viral synapse may play an important role in cell-associated virus entering into epithelial cells [reviewed in [12] ], a phenotype not possible with cell free virus. Bobardt *et al*. reported the rate of transcytosis of cell-free HIV-1 through primary genital epithelial cells to be less than 0.02% of the initial inoculum [8]. Despite the low rate of transcytosis of cell-free virus, both cell-associated and cell-free viruses found in semen have been documented to initiate HIV-1 infection [5], [13]–[16]. However, an animal model study has shown that cell associated virus is harder to cause infection in vivo, further implicating the importance of cell-free virus in HIV-1 transmission [17]. Theoretically, it only takes one infectious viral particle to successfully cross the epithelium to initiate systemic infection [18], [19]; therefore, the passage of cell-free virus via transcytosis as a possible mode of facilitating HIV-1 transmission should not be ignored.

Transcytosis in many cell types, including epithelial cells, is linked to the endocytic recycling pathway [2], [20]. Genital epithelial cells do not express the classical HIV-1 CD4 receptor, making them an unlikely target for viral entry. However, alternate receptors have been implicated in facilitating the attachment of HIV-1 to vaginal epithelial cells [21]–[23]. Stoddard *et al*. demonstrated that gp340, a human scavenger receptor, expressed on vaginal epithelial cells can serve as an HIV-1 receptor to promote trans-infection of HIV-1 susceptible cells [21]. Cargos that are internalized by the myriad of endocytic pathways converge in the early endosome (EE) [24], [25]. From the EE, the endocytosed cargo can be recycled back to the plasma membrane directly or indirectly after traveling to the recycling endosome [24], [25]. Non-recycled cargos are shuttled to the late endosome (LE) and lysosome for degradation [24], [25]. Currently, no mechanism has been established to explain the rare and variable amounts of initial HIV-1 inoculum that are transcytosed as opposed to the majority of viral particles that are not. To date, the precise intracellular pathway that HIV-1 employs to get across vaginal epithelial cells has not yet been completely explored.

In this study, we aimed to determine the intracellular pathway that HIV-1 utilizes to cross the vaginal epithelial cell layer. By use of pharmacological agents, which specifically block each of the recycling or lysosomal pathways, and a dominant negative Rab-11 mutant, we were able to determine the fate of endocytosed HIV-1 in the VK2/E6E7 vaginal epithelial cell line (VK2). Notably, we were able to pinpoint the pathways taken by the virus by viewing the differential trafficking patterns of heat-inactivated viral particles compared to that of native virus. In the absence of specific signals required for HIV-1 loaded EE to be directed into the recycling pathway, virion cargos are degraded via lysosomes. We highlight the possibility of using agents that disrupt signals for sorting of EE into the recycling pathway as a biomedical prophylaxis to inhibit HIV-1 transmission across mucosal epithelia.

## Results

### Native and heat-inactivated HIV-1 enters vaginal epithelial cells at similar efficiencies

**Figure 1:**
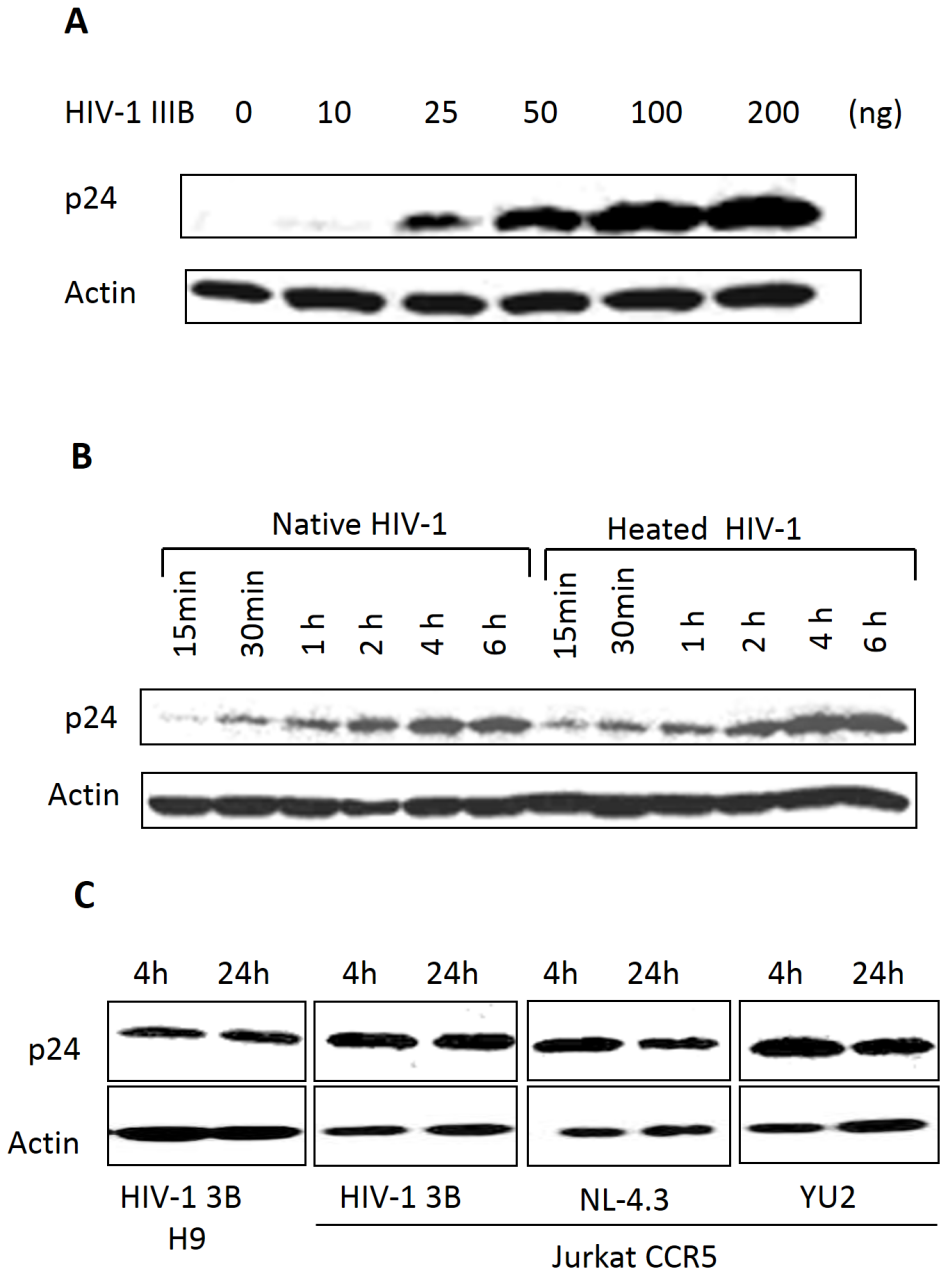
Native and heat-inactivated HIV-1 enters vaginal epithelial cells in a dose- and time-dependent manner. VK2 cells were incubated at 37°C, 5% CO_2_ with indicated amounts of native or heat-inactivated HIV-1, and then the cells were thoroughly washed with PBS and incubated with 0.05% trypsin for 3 min at room temperature to ensure removal of non-internalized virus. (A) HIV-1 IIIB uptake in VK2 cells after exposure to increasing amounts of virus for 4 h was analyzed by Western blot using a p24 antibody with actin staining as the loading control. (B) Time course of viral uptake in vaginal epithelial cells after exposure to 100 ng HIV-1 IIIB. (C) Viral uptake of equal amounts (100 ng) of HIV-1 IIIB obtained from H9 cells or HIV-1 IIIB, YU2 and NL4-3 obtained from Jurkat CCR5 cells. Each experiment in this and subsequent figures was performed at least three times, and representative blots are presented.

An intact epithelial cell layer is efficient in preventing passage of HIV-1; however, the virus is still able to bypass an intact epithelial layer en route to causing systemic infection [7], [8], [26]. It has been argued that cell-free virus is unable to enter epithelial cells [27], [28]. However, when we inoculated VK2 cells with increasing amounts of HIV-1, we found that HIV-1 entered vaginal epithelial cells in a dose- (Fig. 1A) and time- (Fig. 1B) dependent manner with no discernable increase after the 4 h time point. In this experiment, DEAE-dextran was used to enhance the viral uptake, however viral uptake still occurred in the absence of DEAE-dextran (Fig. S1). Consistent with previous reports in different cell types [29]-[32], when VK2 cells were pretreated with a cocktail of increasing amounts of endocytic inhibitors colchicine and dynasore; reduced amounts of HIV-1 were detected inside the cells (Fig. S2). When VK2 cells were exposed to heat-inactivated HIV-1, a similar intracellular accumulation of the heat-inactivated virus occurred compared to that of native HIV-1(Fig. 1B). We also tested the endocytosis of different tropisms of HIV-1 in vaginal epithelial cells. As shown in Figure 1C, HIV-1 IIIB produced from H9 cells and Jurkat-CCR5 cells, as well as the NL4-3 and YU2 virus from Jurkat-CCR5, entered vaginal epithelial cells at similar efficiencies. Our data highlights the fact that vaginal epithelial cells could endocytose CXCR4 and CCR5 virus with equal efficiency as well as virus propagated from different cell types.

### Heat-inactivation of HIV-1 does not affect viral uptake but inhibits viral release from vaginal epithelial cells

**Figure 2:**
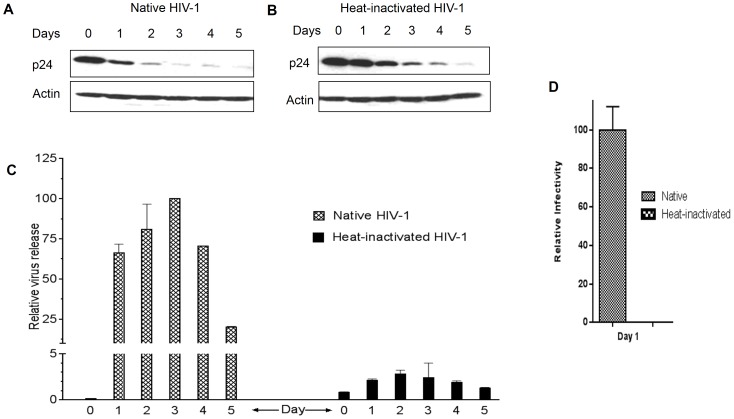
Inactivation of HIV-1 does not affect viral uptake but inhibits viral release from vaginal epithelial cells. VK2 cells were incubated at 37°C, 5% CO_2_ with 100 ng native or heat-inactivated HIV-1 IIIB for 4 h. Cells were then thoroughly washed with PBS and incubated with 0.05% trypsin for 3 min at room temperature to ensure removal of non-internalized virus. Fresh media was added to individual wells, and virus-containing supernatants and cells were harvested at the indicated time points. Viral uptake in cells was analyzed by Western blot (A, B). Virus release in culture supernatants was analyzed by qRT-PCR (C). MAGI assay as used to determine viral infectivity (F) of virus found in supernatant from Day 1. Values are means ± SEM of three independent experiments.

We sought to determine the fate of HIV-1 following uptake by vaginal epithelial cells. VK2 cells were inoculated with native or heat-inactivated HIV-1 for 4 h. After washing and trypsinizing, fresh media was added to the cells. At the indicated time points, cells and supernatants were harvested to measure intracellular and extracellular viral levels respectively. While native and heat-inactivated virus both enter vaginal epithelial cells (Fig. 2A, B), the release of native virus was almost 50-fold more efficient than that of heat-inactivated virus (Fig. 2C). Compared to native HIV-1, only a minute amount of heat-inactivated virus was released from the cells, resulting in a greater intracellular accumulation of p24 (Fig. 2B). We were unable to detect any de novo viral replication in VK2 cells after exposure to HIV-1. The lack of viral Pr55 accumulation over time (Fig. S3) and the inability of AZT to affect virus release (Fig. S3) suggest that there is no detectable de novo viral production. We also tested infectivity of the virus released from the VK2 cells using a viral infectivity assay. VK2 cells were incubated with native or heat-inactivated HIV-1 IIIB for 4 h. Cells were then thoroughly washed with PBS and incubated with 0.05% trypsin for 3 min at room temperature to ensure removal of non-internalized virus. Fresh media was added to individual wells, and virus-containing cell culture supernatants were harvested after 24 h and used to infect TZM-bl indicator cells to measure viral infectivity. Viral inputs were normalized by qRT-PCR. It has been suggest that the infectivity of transcytosed virus is dependent on the cell type it passes through [33], however, our data shows that virus endocytosed by VK2 cells behaved similar to most previous reports [6], [8], [10], [26], [27], [29] which showed that transcytosed virus remains infectious upon release (Fig. 2D). While the exact intracellular pathways that the virus exploits remains unknown, our data suggests that following endocytosis, different intracellular pathways are utilized by the native and heat-inactivated virus and this differential trafficking will be used as a tool to identify the precise pathway that the native virus uses

### Heat-inactivated virus is excluded from the tubulation-dependent endocytic recycling pathway

**Figure 3:**
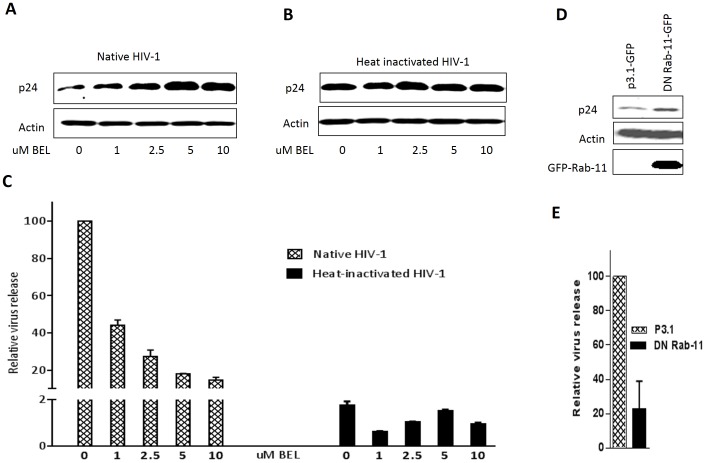
Native HIV-1 utilizes the tubulation-dependent endocytic recycling pathway. VK2 cells were incubated at 37°C, 5% CO_2_ with 100 ng native or heat-inactivated HIV-1 IIIB for 4 h and then thoroughly washed and incubated with 0.05% trypsin to remove non-internalized virus. Fresh media was added to cells with increasing amounts (0, 1, 2.5, 5, 10 uM) of BEL. Cells and supernatants were collected 24 h later for analysis. Intracellular viral protein content was analyzed by Western blot (A, B). Viral release was measured by qRT-PCR (C). VK2 cells were transfected with plasmids expressing DN Rab11-GFP or pCDNA 3.1-GFP as control. Cells were then inoculated with 100 ng HIV-1 IIIB 48 h post-transfection. After washing away the non-internalized inoculum, fresh media was then added. On day 2, levels of intracellular virus and released virus in the supernatant were analyzed using Western blotting (D) and qRT-PCR (E), respectively, as described in Materials and methods. Values are means ± SEM of three independent experiments.

(*E*)-6-(bromomethylene) tetrahydro-3-(1-naphthalenyl)-2H-pyran-2-one (BEL) inhibits endosome tubulation and thus is able to inhibit trafficking from the sorting and recycling endosome [34]. Therefore, we employed this biochemical approach to elucidate the pathways preferred by HIV-1 following endocytosis. After trypsinizing and washing away the initial non-internalized HIV-1 inoculum from VK2 cells, BEL was added at indicated concentrations. Cells and supernatants were harvested after 24 h to determine intracellular and viral release levels, respectively. Treatment of vaginal epithelial cells with 1 µM of BEL reduced viral release by 50% (Fig. 3C). A greater reduction of viral release was observed when increasing concentrations of BEL were added (Fig. 3C). As expected, an accumulation of virus within the cells correlated with impaired viral release (Fig. 3A). When heat-inactivated virus was used, the amount of virus inside the cells remained constant irrespective of the presence of BEL (Fig. 3B), and viral release was unaffected (Fig. 3C). These data suggests that heat-inactivated virus is excluded from the recycling pathways. We tested the cytotoxicity effect of BEL on VK2 cells. No significant cytotoxic effect was defected when up to 2.5 µM BEL was used to treat VK2 cells (Fig. S4). Rab-11 plays a key role in endosome tubulation and is known to associate primarily with recycling endosomes, regulating exocytosis of recycled vesicles [35]. Over-expression of a dominant negative Rab-11 GTPase mutant reduced the release of native HIV-1 from vaginal epithelial cells (Fig. 3E) and slightly increased the accumulation of virus intracellularly (Fig. 3D), corroborating our biochemical results obtained using BEL (Fig. 3A, C).

### Both native and heat-inactivated virus enters the lysosomal degradation pathway

**Figure 4:**
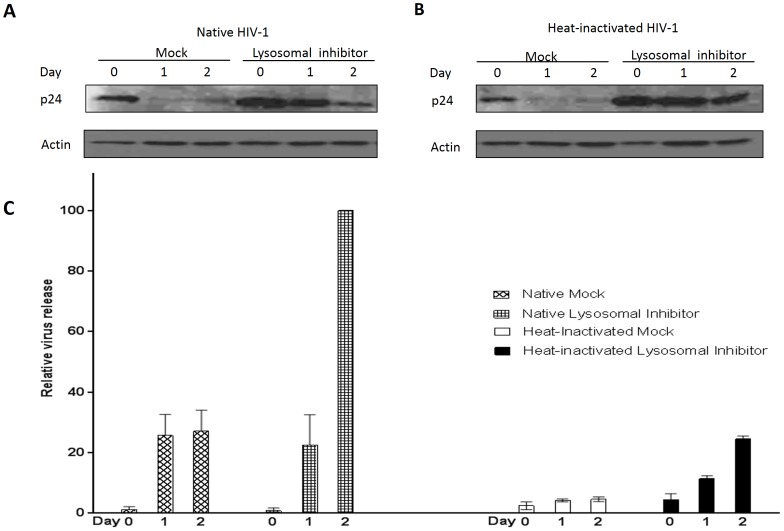
Both native and heat-inactivated virus enter the lysosomal degradation pathway. VK2 cells were pretreated with a cocktail of lysosomal inhibitors (final concentration: 29 µM pepstatin A, 52 µM leupeptin and 69 µM E-64) for 32 h at 37°C, 5% CO_2_. Cells were then inoculated with native or heat-inactivated HIV-1 for 4 h at 37°C, 5% CO_2_ and then washed and trypsinized to remove non-internalized virus. Inhibitors were maintained in culture during the viral exposure. After virus removal, lysosomal inhibitors were added back to the fresh media, and cells and supernatants were collected at indicated time points for analysis by Western blot (A, B) and qRT-PCR (C), respectively. Values are means ± SEM of three independent experiments.

As the heat-inactivated virus was found to be excluded from the recycling pathway (Fig. 3B, C) it was of interest to determine if heat-inactivated virus was being trafficked to the lysosomal degradation pathway. When the lysosomal degradation pathway was blocked with a cocktail of pepstatin A, leupeptin and E-64, and then the cells were inoculated with either native (Fig. 4A) or heat-inactivated (Fig. 4B) virus, an accumulation of virus was observed in lysosomal inhibitor-treated cells compared to mock-treated cells. To our surprise, following treatment with the lysosomal inhibitors, we observed a greater intracellular accumulation of not only the heat-inactivated virus but also the native virus. Interestingly, more heat-inactivated virus accumulated intracellularly compared to native virus indicating that this is the dominant pathway being used by heat-inactivated virus. Blocking the lysosomal degradation pathway lead to a tremendous increase in native viral release on Day 2 (Fig. 4C). There was a slight increase in heat-inactivated viral release as well, however the data clearly shows that heat-inactivated virus is preferentially trafficked to the lysosomal degradation pathway while the native virus is more apt to utilizing alternative pathways (Fig. 4C). This data suggests that while both the native and heat-inactivated virus were able to travel to the lysosomal degradation pathway, the native virus was also able to utilize alternate pathways; meanwhile, the heat-inactivated virus traveled predominantly along the lysosomal route, leading to its higher accumulation in VK2 cells observed following treatment with the lysosomal inhibitor. The trafficking of native virus to the lysosomal pathway may partially explain why only a small amount of the initial amount of HIV-1 inoculum is transcytosed through epithelial cells [8]. Lysosomal inhibitor cocktail used had no cytotoxicity effect to VK2 cells (Fig. S4).

### Transcytosis of HIV-1 is not envelope-dependent

**Figure 5:**
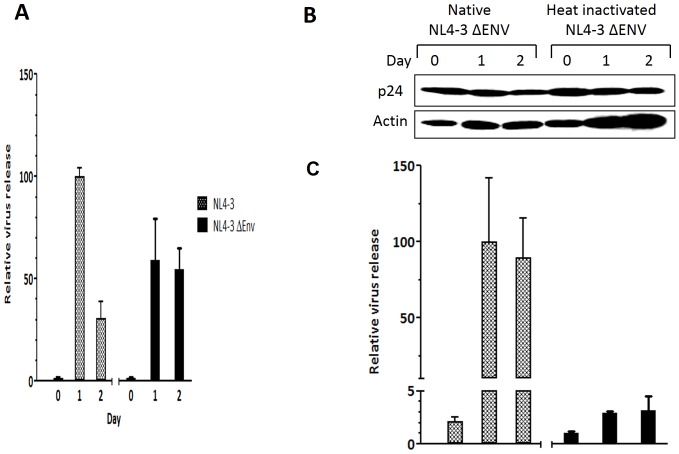
HIV-1 entry into vaginal epithelial cells is envelope-independent, and inhibition of heat-inactivated HIV-1 release is envelope-independent. VK2 cells were incubated for 4-inactivated or untreated NL4-3 or NL4-3 ΔEnv virus. Cells were then thoroughly washed with PBS and incubated with 0.05% trypsin for 3 min at room temperature to ensure removal of non-internalized virus. Fresh media was then added to individual wells, and the supernatants were harvested at indicated time points. Virus release was determined by qRT-PCR (A, C). Intracellular viral protein was analyzed by Western blot (B). Relative virus levels in supernatants were determined using qRT-PCR. Values are means ± SEM of three independent experiments.

The role of HIV-1 envelope for viral entry into epithelial cells remains controversial. While some reports argue that HIV-1 envelope is not needed for entry into epithelial cells [36] [37], others suggest that it is needed [8], [38], [39]. We also tried to address this issue in our system. In our system, wild-type and ΔEnv virus were released from vaginal epithelial cells equally (Fig. 5A), whereas less heat-inactivated ΔEnv virus was released from the cells (Fig. 5C) although similar amounts of native and heat-inactivated ΔEnv virus entered into the VK2 cells (Fig. 5B). This data suggest that HIV-1 enters and exits vaginal epithelial cells in an envelope-independent manner and that the deficiency of heat-inactivated viral release from VK2 cells is dependent on factors on the virus not related to envelope.

### Native HIV-1 is transcytosed using the tubulation-dependent endocytic recycling pathway

**Figure 6:**
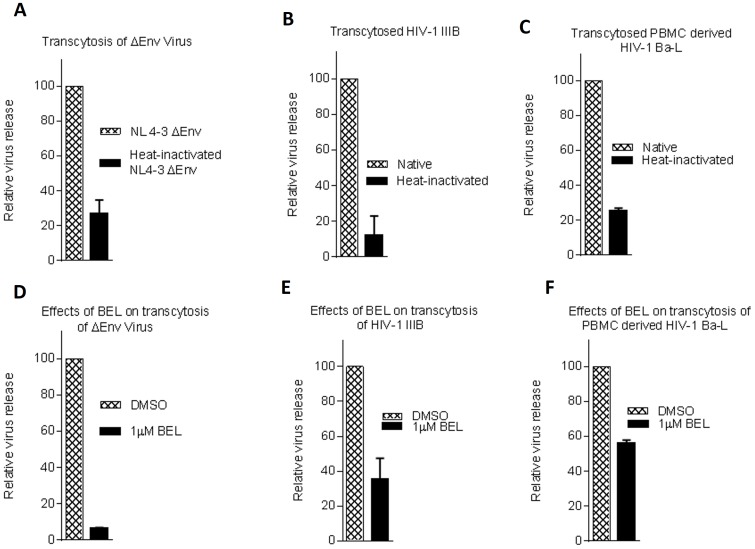
Vaginal epithelial cells support HIV-1 Transcytosis. VK2 cells were grown on a transwell insert containing 3.0 µm pores. Heat-inactivated or untreated NL4-3 ΔENV (100 ng) was then added to the apical chamber and the viral levels in media of the basal chamber were assayed after 1 h incubation at 37°C (A). Native or Heat inactivated HIV-1 IIIB (B) or PBMC derived HIV-1 Ba-L (C) were added to the apical chamber and viral levels in media of the basal chamber were assayed after 1 h. NL4-3 ΔENV (D)HIV-1 IIIB (E) or PBMC derived HIV-1 Ba-L (F) were added to the apical chamber and incubated for 1 h. Media from the apical and basal chambers were removed and replaced with fresh media containing 1 µM BEL. Viral levels in media of the basal chamber were assayed after 24 h using qRT-PCR. Values are means ± SEM of three or more independent experiments

The technique that we have utilized thus far to demonstrate internalization and subsequent viral release have been described and used previously [29]; however, we confirmed our findings using a transwell system, which provides a closer representation of the polarity of these cells *in vivo*. VK2 cells were plated on a 3.0 µm transwell inserts and cultured until fully confluent. Confluencey was confirmed by measuring of the trans-epithelial resistance and lucifer yellow rejection assay. When equal amounts of untreated and heat-inactivated virus were added to the apical chamber; heat-inactivated virus had a diminished capacity to reach the basal chamber using NL4-3 ΔENV (Fig.6A), HIV-1 IIIB produced from H9 cells (Fig. 6B) and HIV-1 Ba-L derived from PBMCs (Fig. 6C). We also tested whether BEL inhibits HIV-1 transcytosis in the transwell system. HIV-1 was added to the apical chamber of the transwell insert containing the vaginal epithelial cells. The virus containing media from the apical chamber and the basal chamber were removed after the incubation period. Fresh media containing BEL was added to both the apical and basal chambers. The culture medium in the basal chamber was collected after 24 h incubation. As shown in Fig. 6, BEL inhibited transcytosis of NL4-3 ΔENV (Fig. 6D), HIV-1 produced in H9 cells (Fig. 6E) and HIV-1 derived from PBMCs (Fig. 6F), indicating that transcytosis of HIV-1 is dependent on intracellular trafficking to the endocytic recycling pathway. Previous studies using transwell inserts coated the transwell insert with collagen and fibronectin to study HIV-1 transcytosis [8]. We repeated our experiments using transwells coated with collagen and fibronectin. As Fig. S5 shows, coating the transwell inserts didn't change the phenotype observed in Fig. 6.

### HIV-1 colocalizes with the recycling endocytic marker transferrin

**Figure 7:**
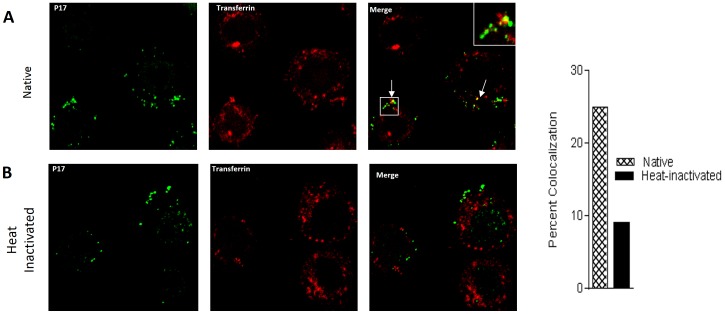
HIV-1 colocalizes with the recycling endocytic marker transferrin. VK2 cells were plated on glass coverslips in a 12-well plate and grown 48 h at 37°C, 5% CO_2_. Native or heat-inactivated HIV-1 IIIB was then added to the cells and incubated for 4 h then subjected to confocal microscopy as described in Materials and methods after removal of non-internalized virus. HIV-1 is shown in green and transferrin is shown in red. Bar graph represents the percentage cells positive for colocalization from the random counting of 162 cells exposed to native and 170 cells exposed to heat-inactivated virus. Arrows points to colocalization signals. Inset is a magnification of the signal that is inside the box.

To further demonstrate the importance of the recycling pathway for HIV-1 transcytosis, we used Immunofluorescence confocal microscopy to visually confirm the presence of HIV-1 in endocytic recycling tubules. Vaginal epithelial cells were grown on coverslips and exposed to native or heat inactivated virus and a fluorescent transferrin conjugate. Transferrin is known to associate with recycling endosomes. Slides were then prepared for confocal analysis using an anti- HIV p17 antibody. As shown in Fig. 7, native virus had a higher rate (26%) of colocalization (indicated by the arrows) with transferrin compared to heat-inactivated virus (9%) (Fig.7). The data further confirmed that native HIV-1, and not heated-inactivated HIV-1 was able to cross vaginal epithelia cell through recycling endocytic pathway.

## Discussion

HIV-1 is transmitted primarily through heterosexual contact [40]. Despite the expansive global spread of HIV-1, the natural transmission of the virus is extremely low [41], [42]. Microtrauma to vaginal tissue increases the risk for HIV-1 acquisition [43]. However, transmission can occur when the mucosal epithelial layer is intact [44], [45]. Langerhans, submucosal dendritic cells and CD4+ T cells are implicated as the first cells in which the virus replicates or by which it is transported during the initial events of transmission [46]–[50]. Vaginal epithelial cells are CD4 negative and express negligible amounts of CXCR4 or CCR5 co-receptors [8] and thus have received minimal attention regarding the initial events of HIV-1 transmission. However, it is important to note that epithelial cells are the most abundant cell type in the vaginal vault and serves as the first line of defense against incoming pathogens. Although an intact epithelial cell layer is extremely robust in preventing the passage of HIV-1, the virus is still able to enter epithelial cells and can be transcytosed to regions where it can come in contact with susceptible cells en route to causing systemic infection. In the current study, we investigated the intracellular pathway utilized by endocytosed HIV-1 to migrate across vaginal epithelial cells. A detailed analysis of the intercellular route taken by the virus once inside vaginal epithelial cells has not been carried out and the mechanism(s) for the small amount of the initial inoculum that travels through vaginal epithelial cells also has not been explored adequately.

In this study, pharmacological agents were used to block the recycling and lysosomal pathway in vaginal epithelial cells to determine the intracellular trafficking of native and heat-inactivated HIV-1. HIV-1 was shown to enter vaginal epithelial cells in a dose- and time-dependent manner (Fig. 1). In replication studies, heat-inactivated virus is commonly used as a replication-incompetent control virus. In the context of transcytosis, however, we demonstrated that heat-inactivated virus enters vaginal epithelial cells at the same rate as native virus (Fig. 1B). However, unlike native virus, heat-inactivated viral release was significantly compromised (Fig. 2, 5). BEL, DN Rab-11 and lysosomal inhibitors enabled us to examine the intracellular pathways utilized by HIV-1 for trafficking in vaginal epithelial cells. BEL inhibits endosome tubulation and at high (10 µM) concentrations inhibits trafficking of endocytosed material from peripheral early/sorting endosomes to central recycling endosomes, while lower (1 µM) concentrations of BEL inhibit trafficking from the central recycling endosomes to the cell surface [34]. Our data suggest that from the pool of virus destined to be transcytosed, once inside vaginal epithelial cells, some virus utilize the rapid recycling pathway from the sorting/early endosome, while the majority of the virions are shuttled to the recycling endosome and then exit the cells (Fig. 3). Pinpointing the exact pathways that the virus uses once inside vaginal epithelial cells, along with using a transwell system (Fig. 5) and demonstrating viral colocalization with recycling endosomes (Fig. 6), we provide conclusive evidence that vaginal epithelial cells support cell-free viral transcytosis. We also our model to test whether cell-associated virus uses the same intracellular pathway as cell free virus did. As demonstrated in Fig. S6, BEL was also able to inhibit transcytosis of cell-associated HIV-1, suggesting that cell-associated HIV might also get across VK2 cells through the recycling endosomal pathway.

Numerous studies using cervical, intestinal and other epithelial cell types have shown that HIV-1 is able to get across an intact epithelial cell layer by transcytosis [6]–[10]. Maher et al used confocal microscopy combined with 3D surface reconstruction to demonstrate that HIV-1 could both bind to the external surface of epithelial cells and actually penetrate beneath the epithelial surface in a cervicovaginal organ tissue culture system [51], which supports the idea that HIV-1 can cross the vaginal epithelial layer through either transcytosis or through the junctions between the epithelial cells. A recent finding has suggested that vaginal epithelial cells may play a bigger role in HIV-1 acquisition than previously believed and argued that the virus is passing between the cells through a diffusive percolation mechanism, penetrating through areas where junctions are absent [52]. Our work demonstrates, for the first time, the precise trafficking pathways that HIV-1 utilizes following entry into vaginal epithelial cells. We show that the virus is able to pass directly through epithelial cells and treatment of vaginal epithelial cells with BEL or expression of a Rab-11 dominant negative mutant blocked this process. Knowing that the virus interacts with epithelial cells in a human cervicovaginal organ tissue culture system [51] and demonstrating the pathways that HIV-1 exploits once it comes in contact with vaginal epithelial cells, shows that transcytosis of HIV-1 through vaginal epithelial cells does occur and provides an additional mechanism along with diffusive percolation reaffirming the importance of cells from the lower female reproductive tract in HIV-1 acquisition.

Heat inactivated HIV-1 has been well characterized and is commonly used to produce replication-incompetent virus [53]–[55]. Previous studies have demonstrated that heat inactivation of HIV-1 leads to the shedding of gp120 but leaves the viral membrane intact [53]-[55]. Although it is enticing to think that removal of gp120 from the heat-inactivated virus prevents it from entering the recycling pathway and thus inhibits its release from vaginal epithelial cells, our data suggests that transcytosis of HIV-1 is envelope-independent (Fig. 5). The role of HIV-1 envelope for viral entry into epithelial cells remains controversial. While some reports argue that HIV-1 envelope is not needed for entry into epithelial cells [36] [37], others suggest that it is needed [8], [38], [39]. Our experiment demonstrated that HIV-1 could enter vaginal epithelial cells without envelope, aiding in the conclusion that a factor other than envelope plays a role in the differential trafficking of native and heat-inactivated virus. The mechanism warrants further investigation.

New HIV-1 infections are largely the result of sexual contact. Since HIV-1 virions and HIV-1 infected cells are present in the semen and cervical mucus of infected individuals, HIV-1 prevention strategies must consider both cell-free and cell-associated virus. In this study, we not only demonstrate the intracellular route used by HIV-1 for transcytosis, we also demonstrate that cell-free heat-inactivated HIV-1 particles were unable to be released from vaginal epithelial cells even though they had no problems entering. This was unexpected as heat inactivation of HIV-1 is commonly used to produce replication-incompetent virus that is inhibited at the entry step of the viral lifecycle. It is clear; however, that uptake of HIV-1 by vaginal epithelial cells is not affected by heat inactivation (Fig. 1, 2,3,4,5). We are currently evaluating what causes the differential trafficking of heat-inactivated virus.

## Conclusions

HIV-1 is able to enter vaginal epithelial cells through endocytosis and subsequently make use the cell's recycling pathways to facilitate transcytosis of HIV-1. Our work demonstrates, for the first time the precise trafficking pathways that HIV-1 utilizes following entry into vaginal epithelial cells and shows that heat-inactivated virus enters vaginal epithelial cells with the same efficiency as native HIV-1 but is trafficked differently once inside the cells.

## Methods

### Cell culture

Human embryonic kidney (HEK) 293T, human T cell line H9 and TZM-bl cells were obtained from the NIH AIDS Research and Reference Reagent Program (NIH-ARRRP) and were cultured in regular cultural medium containing 10% fetal bovine serum (FBS) at 37°C and 5% CO_2_. The human Jurkat T cell line stably expressing CCR5 (Jurkat-CCR5) [56] was a gift from Dr. Michael Emerman (University of Washington). The VK2/E6E7 (ATCC CRL-2616) cell line was established from the normal vaginal mucosal tissue of a premenopausal woman undergoing anterior-posterior vaginal repair surgery and has maintained expression of tissue-specific differentiation proteins [57]. VK2 cells have been used successfully in numerous studies to faithfully mimic the lower female reproductive track epithelial barrier [23], [58]–[61]. VK2 cells were cultured in keratinocyte serum-free medium (Life Technologies, Gibco BRL) supplemented with 50 µg/mL of bovine pituitary extract, 0.1 ng/mL of epidermal growth factor and 0.4 mM CaCl_2,_ at 37°C and 5% CO_2_. VK2 cells (3×10^5^) were plated in 12-well-plates and allowed to grow until fully confluent unless otherwise indicated.

### Plasmids, transfection, Western blot, antibodies and reagents

HIV-1 proviral constructs pNL4-3 [62], and pYU2 [63] along with zidovudine (AZT)were obtained from the NIH-ARRRP. Transfection of the dominant negative Rab11 mutant (DN Rab11, Addgene) was performed using Lipofectamine 2000 (Invitrogen) following the manufacturer's manual. Western blot analysis was carried out as previously described [64]. Colchicine, dynasore hydrate, DEAE-dextran hydrochloride and (*E*)-6-(bromomethylene) tetrahydro-3-(1-naphthalenyl)-2H-pyran-2-one (BEL) were purchased from Sigma. Lysosomal inhibitors pepstatin A, leupeptin and E-64 were purchased from AG Scientific. anti-GFP antibody was purchased from Santa Cruz Biotechnology, Inc. The HIV-1 p24 monoclonal antibody [65] was obtained from NIH-ARRRP. Transferrin was purchased from Invitrogen and staining was performed using the manufacturer's protocol.

### Virus preparation

HIV-1 IIIB virus was obtained from the supernatant of HIV-1 IIIB chronically infected H9 cells [66]. HIV-1 Ba-L was obtained from NIH-ARRRP. To generate NL4-3, NL4-3ΔENV or YU2 virus, 293 T cells were transiently transfected with pNL4-3, pNL4-3-deltaE-EGFP and pYU2 plasmids respectively using polyethylenimine (PEI) as described previously [67]. The culture supernatants were used to infect Jurkat-CCR5 cells to establish chronically infected cell lines. HIV-1 IIIB, NL4-3 or YU2 virus was obtained by harvesting the supernatants of these chronically infected Jurkat-CCR5 cell lines. To generate virus from primary cells, PHA activated PBMCs were infected with HIV-1 IIIB or HIV-1 Ba-L. Virus was obtained by harvesting the supernatants of the infected PBMCs. Viral heat inactivation [53] were performed as previously described. Heat inactivated along with all other viral preparations were subjected to ultra-centrifugation at 100,000×*g* for 2 h on a 20% sucrose cushion and normalized before use with a p24 ELISA kit (AIDS and Cancer Virus Program, National Cancer Institute at Frederick) or a quantitative real-time PCR (qRT-PCR) assay using the primers LTR S4 (AAGCCTCAATAAAGCTTGCCTTGA) and LTR AS3 (GTTCGGGCGCCACTGCTAG) as described previously [68]. The only modification in the qRT-PCR assay was that SYBR Green was used instead of a 6-carboxy-fluorenscein labeled probe as mentioned in a previous study [68].

### HIV-1 infection/endocytosis/viral release assay

VK2/E6E7 cells in a 12-well-plate format were incubated with indicated amounts of virus mixed with 20 µg/ml DEAE-dextran hydrochloride to facilitate attachment for the indicated amount of time. Cells were then thoroughly washed with PBS and incubated with 0.05% trypsin for 3 min at room temperature to ensure removal of non-internalized virus following the method previously described [8], [29]. Trypsin was inactivated with DMEM containing 10% FBS, followed by washing of the cells with PBS at least 3 times. Fresh media was then added to individual wells, and the supernatants and cells were harvested at the indicated time points. Increasing amounts of a combination of endocytic inhibitors were added 30 min (cholchine) and 1 h (dynasore) before addition of virus. The tubulation inhibitor BEL was added at indicated concentrations to fresh media immediately after the virus was removed from cells. Twenty-four hours later, cells and supernatants were harvested for Western blot and qRT-PCR analyses, respectively. A cocktail of lysosomal inhibitors (final concentration: 29 µM pepstatin A, 52 µM leupeptin and 69 µM E-64) was added 32 h prior to and during exposure of VK2 cells to HIV-1 Inhibitors. After virus removal, lysosomal inhibitors were added back to the fresh media, and cells and supernatants were collected at indicated time points for Western blot and qRT-PCR analyses, respectively. MAGI assay were carried out as previously described [67].

### Cell Immunofluorescence staining and confocal microscopy analysis

Confocal microcopy analysis was performed as described previously [64], [69] with a few modifications. Briefly, VK2 cells were plated on glass coverslips in a 12-well plate and grown 48 h, 70% confluence. Native or heat-inactivated HIV-1 IIIB was then added to the cells and incubated for 4 hrs. Transferrin (3 uM) was added 1 hr and 30 mins respectively before the end of the 4 hrs viral incubation. Cells were then thoroughly washed with PBS and incubated with 0.05% trypsin for 3 min at room temperature to ensure removal of non-internalized virus. Trypsin was inactivated with DMEM containing 10% FBS, followed by washing of the cells with PBS. Cells were then fixed with 4% paraformaldehyde in PBS solution at room temperature for 10 minutes, permeabilized with 0.1% Triton X-100 for 10 minutes, and blocked with 5% bovine serum albumin overnight. Immunofluorescent staining was performed using polyclonal anti–HIV-1 p17 (VU47) as primary antibodies, which were detected via goat anti-rabbit Alexa 488–conjugated secondary antibodies. Images were captured using a Nikon A1R confocal microscope.

### Transwell system

Model transwell assays has been previously described [70]. Briefly, to establish an intact vaginal epithelial layer, VK2 cells were plated (1.5×10^5^) and grown in a 6.5-mm diameter transwell in a 24-well plate on a transwell polycarbonate membrane containing 3.0 µm pores (Corning). Cells were allowed to grow into a tight, polarized apical and basolateral surfaces. Integrity of epithelial layer was monitored by measuring transepithelial electrical resistance with a volt-ohm meter (Millipore Millicell-ERS, Millipore, Concord, MA), and monolayers were used only when the transepithelial electrical resistance was 400 mΩ/cm^2^ or greater, consistent with non-permeable, intact tight junctions. Similar monolayers were used in other studies using primary vaginal epithelial cells [8]. Lucifer Yellow rejection assay [71] was also performed at the end of each experiment to confirm that the monolayer was still intact. Indicated virus (100 ng) was added to the apical chamber of the transwell insert. The viral levels in media of the basal chamber were assayed after 1 h. For BEL experiments, native 1 IIIB, Env-deficient virus or PBMC derived HIV-1 Ba-L were added to the apical chamber and incubated for 1h. Media from the apical and basal chambers were removed and replaced with fresh media containing 1 µM BEL. Viral levels in media of the basal chamber were assayed after 24 h.

## Supporting Information

Figure S1 **HIV-1 enters VK2 cells without DEAE-dextran but is enhanced with DEAE-dextran.** VK2 cells were incubated at 37°C, 5% CO_2_ with indicated amounts of native HIV-1 in the absence or presence of DEAE-dextran for 4 h. The cells were thoroughly washed with PBS and incubated with 0.05% trypsin for 3 min at room temperature to ensure removal of non-internalized virus. HIV-1 IIIB uptake in VK2 cells was assayed by Western blot using a p24 antibody with actin staining as the loading control.
(TIF)

Figure S2 **HIV-1 enters VK2 cells through endocytosis.** VK2 cells were incubated at 37°C, 5% CO_2_ with 100 ng native or heat-inactivated HIV-1 IIIB after pretreatment with increasing amounts (0,25,50,100 uM) of colchicine (30 min) combined with increasing amounts (0,25,50,100 uM) dynasore (1 h). Cells were then thoroughly washed with PBS and incubated with 0.05% trypsin for 3 min at room temperature to ensure removal of non-internalized virus. Western blot was performed using a p24 antibody with actin staining as the loading control. The lower panel shows a Western blot of viral inoculum of known concentrations of HIV-1. Western blot was performed using a p24 antibody.
(TIF)

Figure S3 **No detectable HIV-1 replication in VK2.** VK2 cells were incubated at 37°C, 5% CO_2_ with 100 ng HIV-1 IIIB in the presence of 100 uM AZT or DMSO for 6 h. Cells were then thoroughly washed with PBS and incubated with 0.05% trypsin for 3 min at room temperature to ensure removal of non-internalized virus. Fresh media was then added with AZT or DMSO. Culture media was harvested to assay viral levels using qRT-PCR. Center panel demonstrates that AZT was functional as it was able to inhibit replication of HIV-1 in Sup-T1 cells. Western blot analysis of intracellular p24 demonstrates that there is no p55 accumulation over time.
(TIF)

Figure S4 **No appreciable cytotoxic effects of BEL and lysosomal degradation inhibitors VK2 cells.** VK2 cells were mock treated (DMSO) or treated with a cocktail of lysosomal inhibitors (final concentration: 29 µM pepstatin A, 52 µM leupeptin and 69 µM E-64) for 32 h or increasing concentration of BEL for 24 h then harvested and stained by LIVE/DEAD Cell Vitality Assay Kit (Invitrogen). Cells were analyzed on a BD Biosciences FACScalibur, exciting at 488 nm and measuring the fluorescence emission at 530 nm and 575 nm.
(TIF)

Figure S5 **Transcytosis of HIV-1 through VK2 cells plated on collagen and fibronectin coated transwell inserts.** VK2 cells were grown on a transwell insert containing 3.0 µm pores coated with collagen and fibronectin. (Left) Native or Heat inactivated HIV-1 IIIB were added to the apical chamber and viral levels in media of the basal chamber were assayed after 1 h using qRT-PCR. (Right) Media from the apical and basal chambers were removed and replaced with fresh media containing 1 µM BEL. Viral levels in media of the basal chamber were assayed after 24 h using qRT-PCR. Values are means ± SEM of three or more independent experiments
(TIF)

Figure S6 **Cell associated HIV-1 utilizes the tubulation-dependent endocytic recycling pathway.** VK2 cells were grown on a transwell insert containing 3.0 µm pores coated with collagen and fibronectin. H9 cells (5×10^5^) chronically infected with HIV-1 IIIB were added to the apical chamber for 3 h. Inserts were then transferred to new wells containing fresh media with 1 µM BEL. Fresh media containing BEL was also added to the apical chamber. Viral levels in media of the basal chamber were assayed after 1 h using qRT-PCR.
(TIF)
