# Supplementary figures and images for: Transcytosis of HIV-1 through Vaginal Epithelial Cells Is Dependent on Trafficking to the Endocytic Recycling Pathway
Source: PLoS One. 2014 May 15;9(5):e96760. doi: 10.1371/journal.pone.0096760 (PMC4022679; doi:10.1371/journal.pone.0096760)

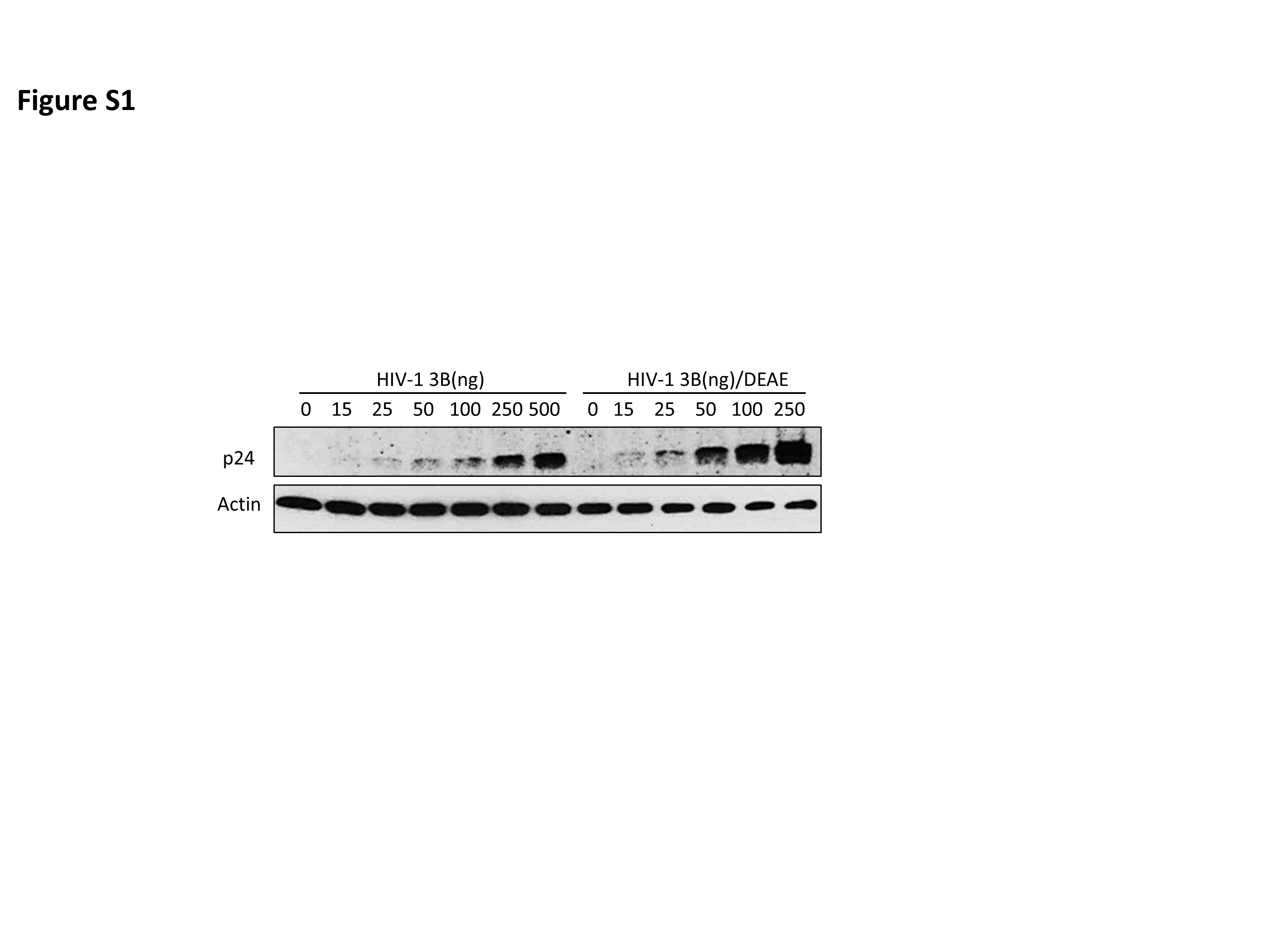

Supplement: Figure S1 — HIV-1 enters VK2 cells without DEAE-dextran but is enhanced with DEAE-dextran. VK2 cells were incubated at 37°C, 5% CO2 with indicated amounts of native HIV-1 in the absence or presence of DEAE-dextran for 4 h. The cells were thoroughly washed with PBS and incubated with 0.05% trypsin for 3 min at room temperature to ensure removal of non-internalized virus. HIV-1 IIIB uptake in VK2 cells was assayed by Western blot using a p24 antibody with actin staining as the loading control. (TIF) [file pone.0096760.s001.tif]

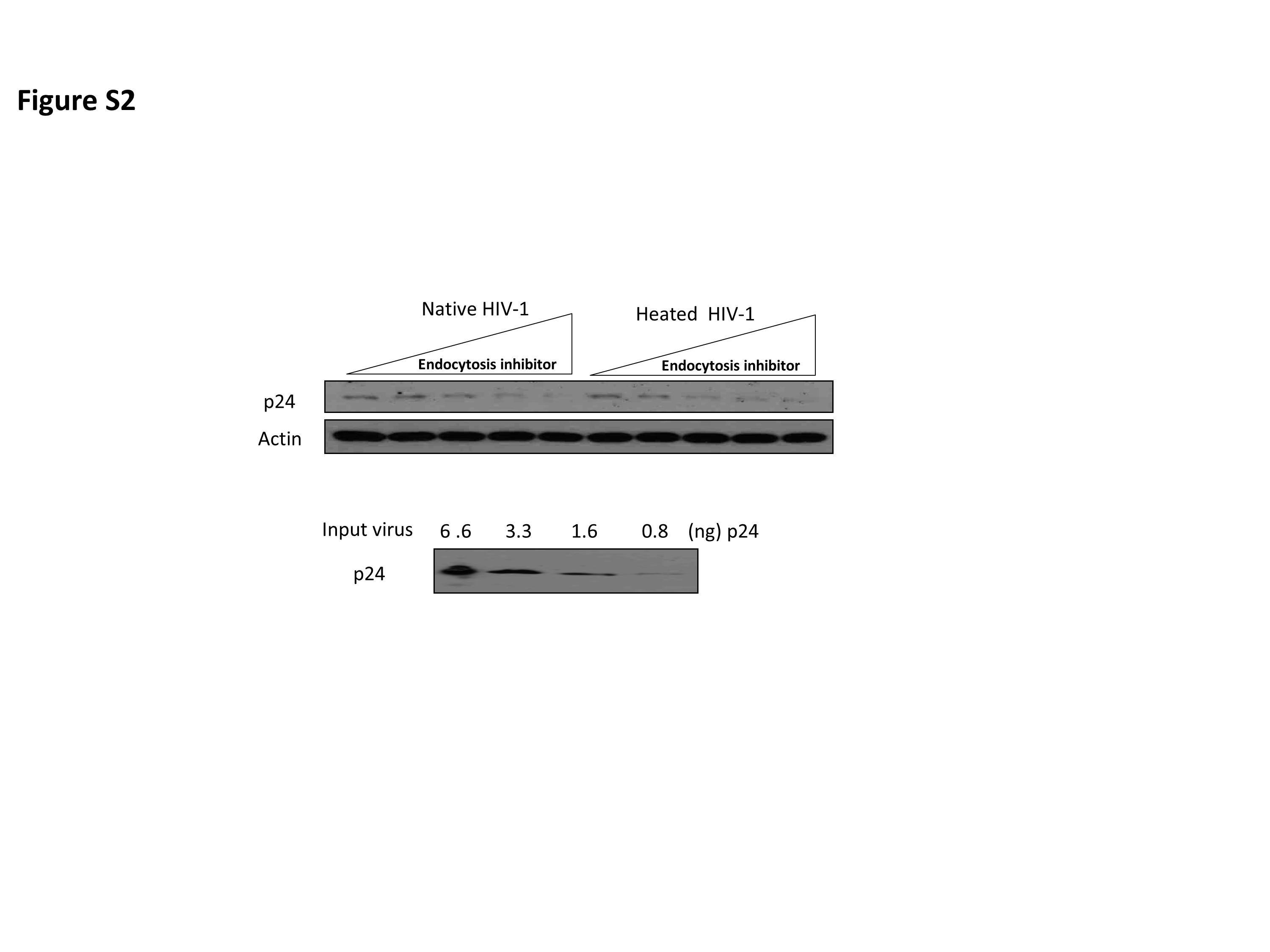

Supplement: Figure S2 — HIV-1 enters VK2 cells through endocytosis. VK2 cells were incubated at 37°C, 5% CO2 with 100 ng native or heat-inactivated HIV-1 IIIB after pretreatment with increasing amounts (0,25,50,100 uM) of colchicine (30 min) combined with increasing amounts (0,25,50,100 uM) dynasore (1 h). Cells were then thoroughly washed with PBS and incubated with 0.05% trypsin for 3 min at room temperature to ensure removal of non-internalized virus. Western blot was performed using a p24 antibody with actin staining as the loading control. The lower panel shows a Western blot of viral inoculum of known concentrations of HIV-1. Western blot was performed using a p24 antibody. (TIF) [file pone.0096760.s002.tif]

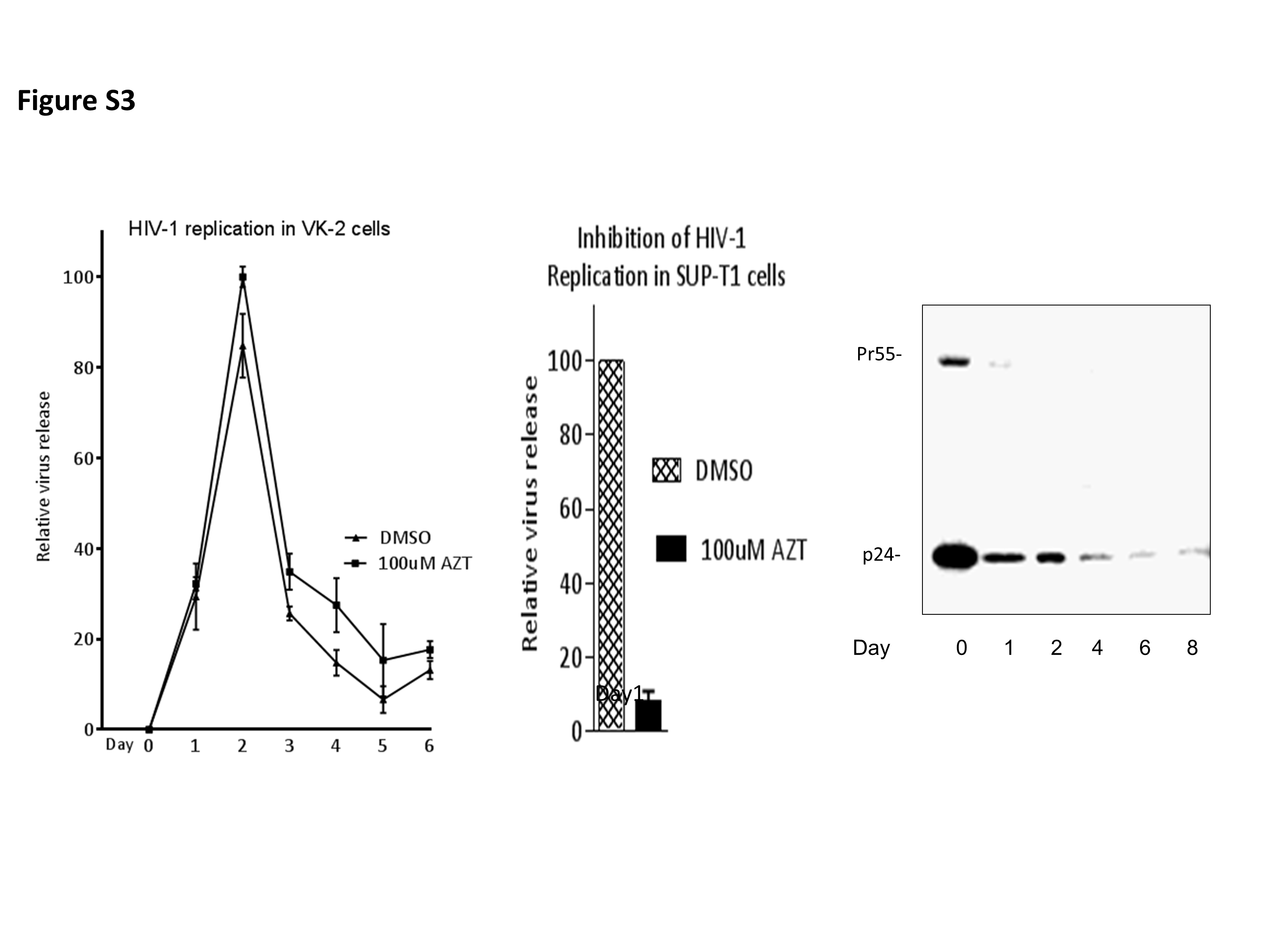

Supplement: Figure S3 — No detectable HIV-1 replication in VK2. VK2 cells were incubated at 37°C, 5% CO2 with 100 ng HIV-1 IIIB in the presence of 100 uM AZT or DMSO for 6 h. Cells were then thoroughly washed with PBS and incubated with 0.05% trypsin for 3 min at room temperature to ensure removal of non-internalized virus. Fresh media was then added with AZT or DMSO. Culture media was harvested to assay viral levels using qRT-PCR. Center panel demonstrates that AZT was functional as it was able to inhibit replication of HIV-1 in Sup-T1 cells. Western blot analysis of intracellular p24 demonstrates that there is no p55 accumulation over time. (TIF) [file pone.0096760.s003.tif]

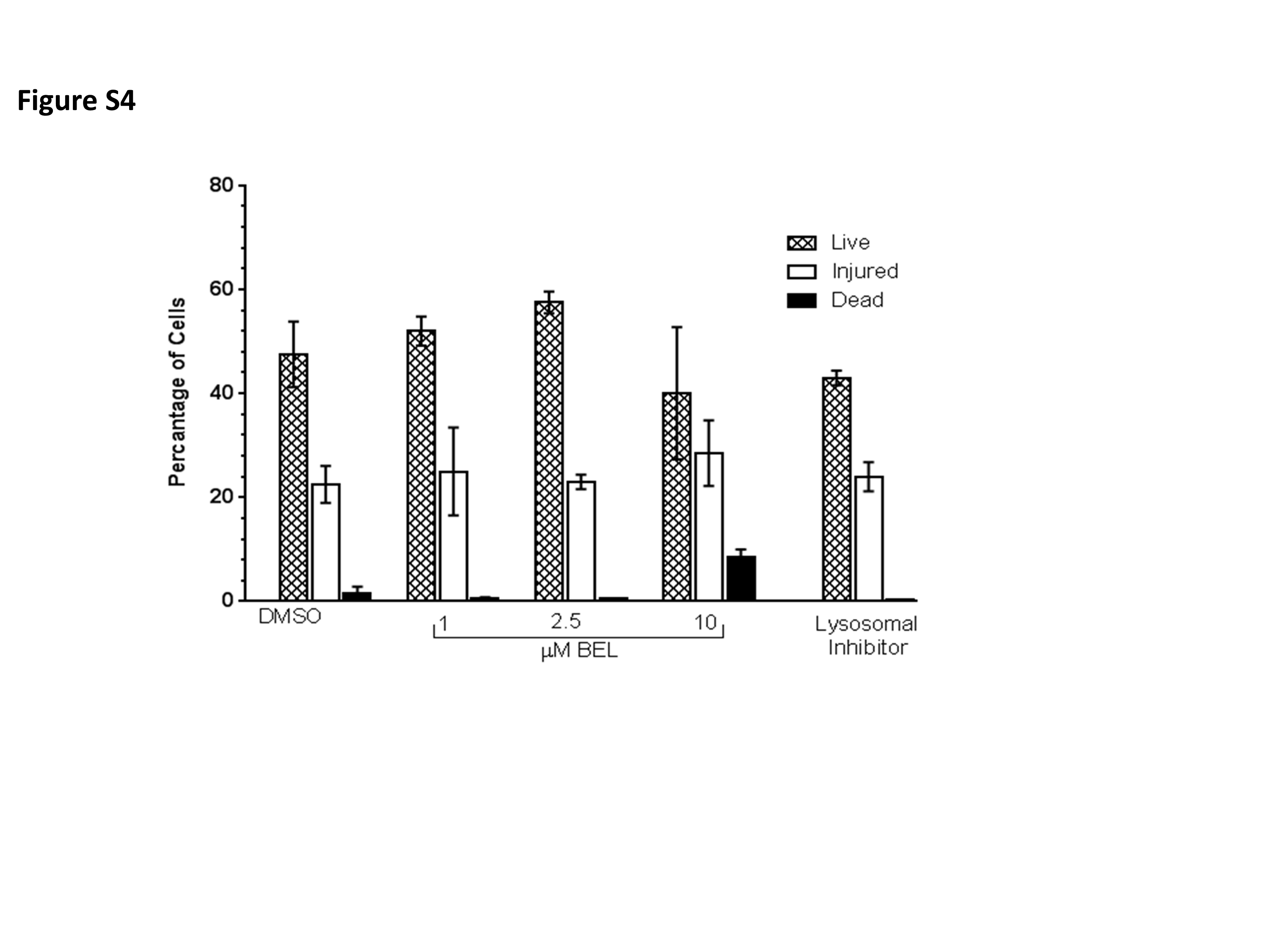

Supplement: Figure S4 — No appreciable cytotoxic effects of BEL and lysosomal degradation inhibitors VK2 cells. VK2 cells were mock treated (DMSO) or treated with a cocktail of lysosomal inhibitors (final concentration: 29 µM pepstatin A, 52 µM leupeptin and 69 µM E-64) for 32 h or increasing concentration of BEL for 24 h then harvested and stained by LIVE/DEAD Cell Vitality Assay Kit (Invitrogen). Cells were analyzed on a BD Biosciences FACScalibur, exciting at 488 nm and measuring the fluorescence emission at 530 nm and 575 nm. (TIF) [file pone.0096760.s004.tif]

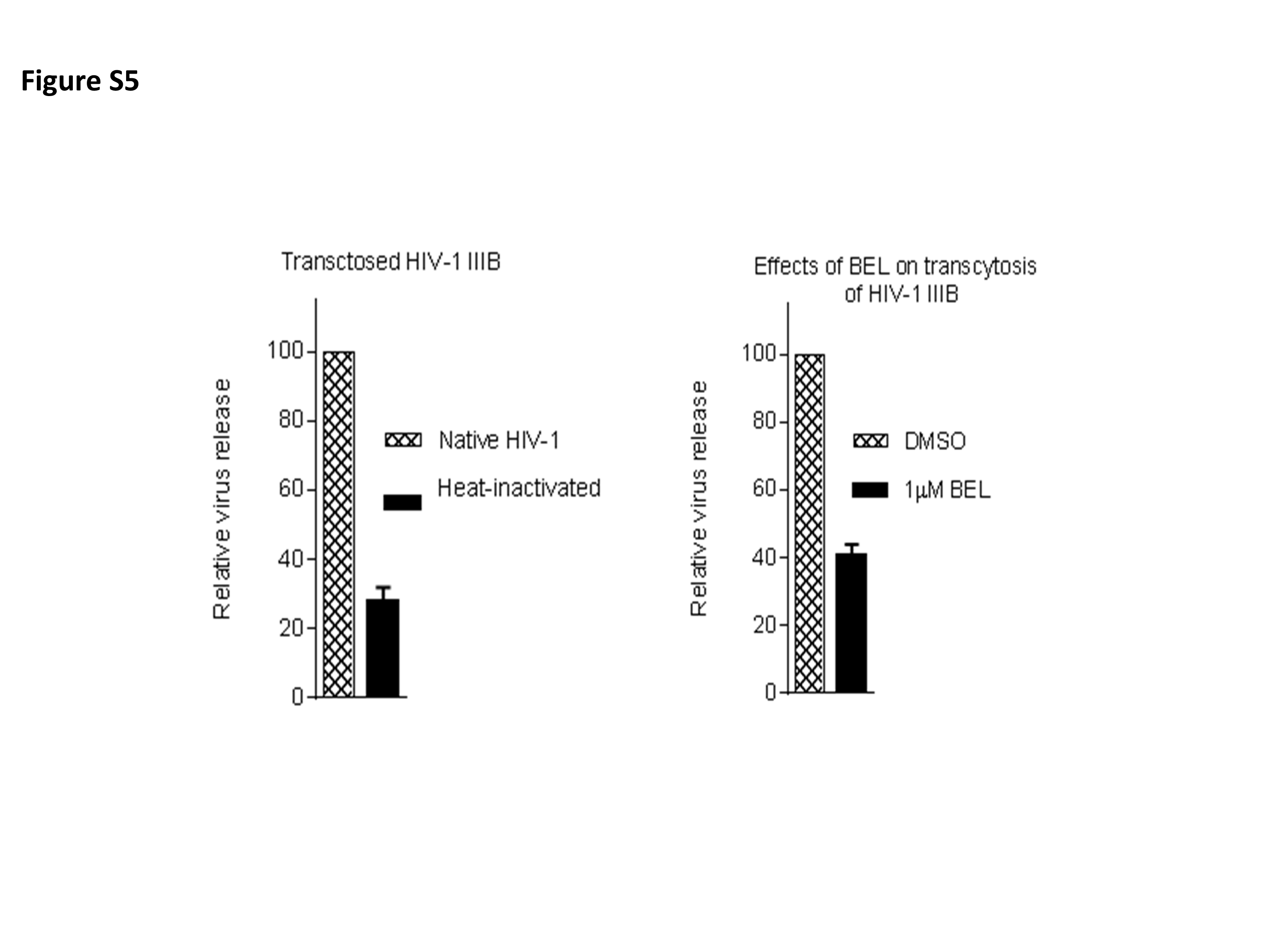

Supplement: Figure S5 — Transcytosis of HIV-1 through VK2 cells plated on collagen and fibronectin coated transwell inserts. VK2 cells were grown on a transwell insert containing 3.0 µm pores coated with collagen and fibronectin. (Left) Native or Heat inactivated HIV-1 IIIB were added to the apical chamber and viral levels in media of the basal chamber were assayed after 1 h using qRT-PCR. (Right) Media from the apical and basal chambers were removed and replaced with fresh media containing 1 µM BEL. Viral levels in media of the basal chamber were assayed after 24 h using qRT-PCR. Values are means ± SEM of three or more independent experiments (TIF) [file pone.0096760.s005.tif]

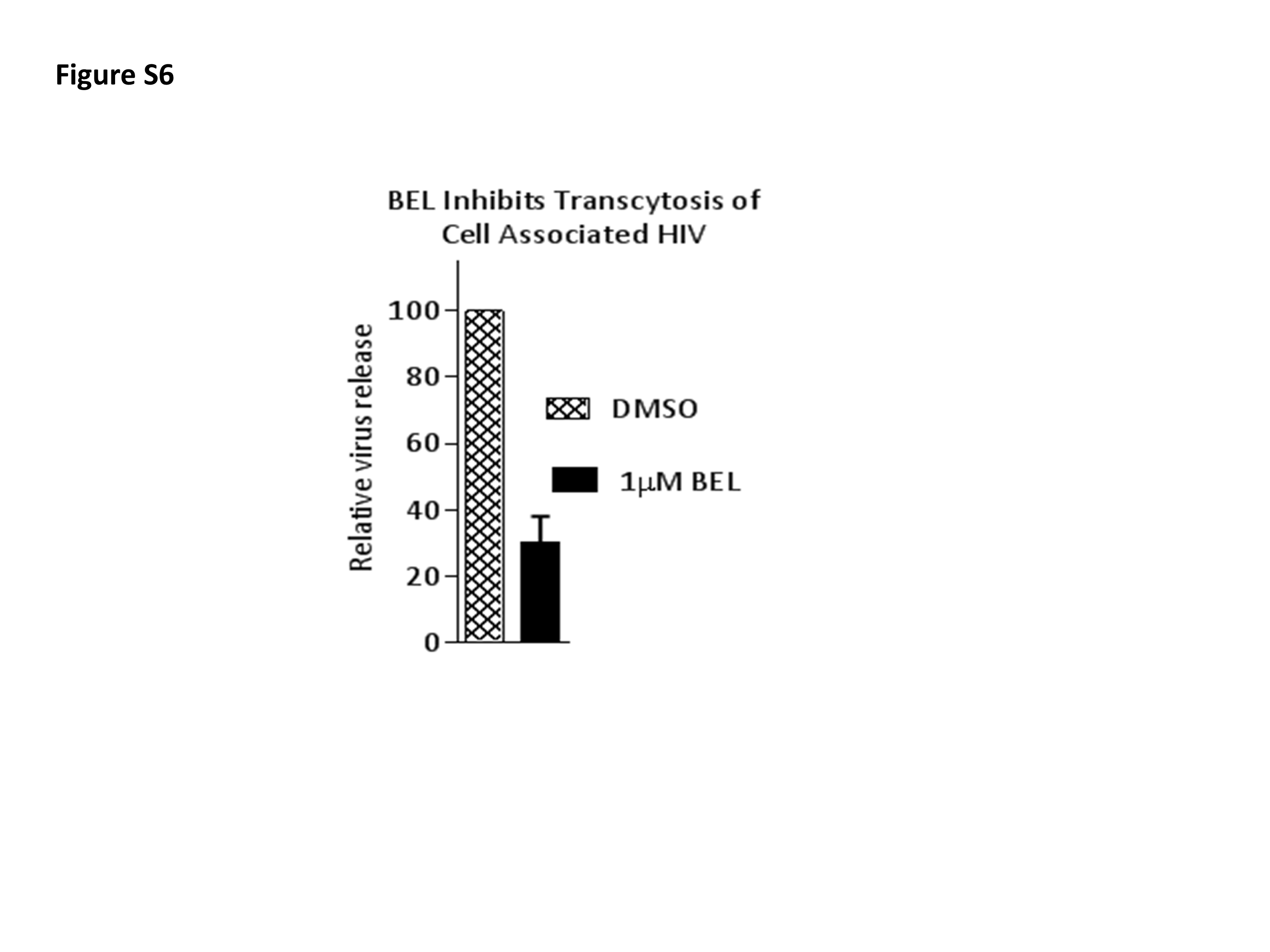

Supplement: Figure S6 — Cell associated HIV-1 utilizes the tubulation-dependent endocytic recycling pathway. VK2 cells were grown on a transwell insert containing 3.0 µm pores coated with collagen and fibronectin. H9 cells (5×10^5^) chronically infected with HIV-1 IIIB were added to the apical chamber for 3 h. Inserts were then transferred to new wells containing fresh media with 1 µM BEL. Fresh media containing BEL was also added to the apical chamber. Viral levels in media of the basal chamber were assayed after 1 h using qRT-PCR. (TIF) [file pone.0096760.s006.tif]
